# Development of a 3D Relative Motion Method for Human–Robot Interaction Assessment

**DOI:** 10.3390/s22062411

**Published:** 2022-03-21

**Authors:** Felipe Ballen-Moreno, Margarita Bautista, Thomas Provot, Maxime Bourgain, Carlos A. Cifuentes, Marcela Múnera

**Affiliations:** 1 Robotics & Multibody Mechanics (R&MM) Research Group, Department of Mechanical Engineering, Vrije Universiteit Brussel, 1050 Brussels, Belgium; felipe.ballen.moreno@vub.be; 2 Department of Biomedical Engineering, Colombian School of Engineering Julio Garavito, Bogota 111166, Colombia; laura.bautista-a@mail.escuelaing.edu.co (M.B.); marcela.munera@escuelaing.edu.co (M.M.); 3 EPF Graduate School of Engineering, F-92330 Sceaux, France; thomas.provot@epf.fr (T.P.); maxime.bourgain@epf.fr (M.B.); 4 Institut de Biomécanique Humaine Georges Charpak, Arts et Metiers Institute of Technology, F-75013 Paris, France; 5 School of Engineering, Science and Technology, Universidad del Rosario, Bogota 111711, Colombia

**Keywords:** exoskeleton, human–robot interaction, relative motion

## Abstract

Exoskeletons have been assessed by qualitative and quantitative features known as performance indicators. Within these, the ergonomic indicators have been isolated, creating a lack of methodologies to analyze and assess physical interfaces. In this sense, this work presents a three-dimensional relative motion assessment method. This method quantifies the difference of orientation between the user’s limb and the exoskeleton link, providing a deeper understanding of the Human–Robot interaction. To this end, the AGoRA exoskeleton was configured in a resistive mode and assessed using an optoelectronic system. The interaction quantified a difference of orientation considerably at a maximum value of 41.1 degrees along the sagittal plane. It extended the understanding of the Human–Robot Interaction throughout the three principal human planes. Furthermore, the proposed method establishes a performance indicator of the physical interfaces of an exoskeleton.

## 1. Introduction

Lower limb exoskeletons (LLEs) are commonly used to enhance and assist different human activities. These devices provide a robotic physical aid, which can be used in different scenarios, such as performing tasks on the treadmill or directly on the floor [1,2]. Within these two scenarios, the main goal of the use of LLEs has often been classified into three categories: (1) rehabilitation, (2) assistance, and (3) augmentation [3,4]. Rehabilitation exoskeletons are aimed to support physical therapy tasks, which require demanding training to provoke neural plasticity, build muscle strength, promote balance, and regain healthy patterns [5]. Similarly, assistance exoskeletons have been designed to assist in activities of daily living, which are also required by the patient, such as stand-to-sit, sit-to-stand, walking up/downstairs, among others [6]. On the other hand, augmentation exoskeletons are focused on enhancing the user’s capabilities, providing sufficient support, and inducing a reduction in user effort [7]. According to the exoskeleton category, LLEs could include different functionalities and features to interact with the user. Here, the assessment of these devices is at the forefront of understanding the user’s interaction.

The LLE’s assessment is performed through multiple users’ motor skills, regardless of their main goal [8,9]. These motor skills are defined through the user’s needs or clinician’s requirements, either enhancement, assistance, or rehabilitation applications [10,11]. Pinto-Fernandez et al. [12] established three main groups divided into walking (i.e., walking on a treadmill, walking on flat ground, walking on slopes, walking on irregular or soft ground, weight-bearing), standing (i.e., standing on moving ground, standing during manipulation, standing during pushes, standing on slopes), and other (i.e., lateral stepping, kneeling, sit-to-stand, running). The LLE is assessed through performance indicators with each motor skill compared to other devices.

These performance indicators are divided into three main groups: goal-level parameters, the user or device kinematics/kinetics along with the trial (e.g., spatiotemporal parameters, joint kinematics or kinetics), and human–robot interaction outcomes (e.g., ergonomics, metabolic costs, internal interaction forces, safety) [12,13]. The first group is the goal-level parameters aimed at the overall task to achieve. They are established by the maximum speed, the minimum time for donning/doffing, the time of the walking test (e.g., 6-m walking test, 10-m walking test), distance, endurance, versatility, and dependability [14,15]. The second group is intended for the user’s kinematics and kinetics commonly used to assess LLEs. Similarly, the device’s indicators are also evaluated through kinematic and kinetic outcomes [12,16,17]. Previous indicators analyzed the joints of interest (e.g., joint moments, joint kinematics, and kinetics) and the spatiotemporal parameters (e.g., cadence, step length, step width, walking speed, phase time), performing a specific task that induces a motor skill. Finally, the last group comprehends the Human–Robot Interaction (HRI) indicators relating device’s ergonomics and comfort, muscle and cognitive effort, interaction forces, and metabolic cost [18,19]. In addition, HRI indicators provide meaningful information to understand intrinsic issues such as soft tissue artefact, intra/inter-subject differences, and joint misalignment [20,21].

The primary device component involved in the above indicators is the physical interface, which constitutes a key component of exoskeletons. They provide force transmission and secure the device to the user [22,23]. Within the HRI indicators, the physical interfaces are assessed through ergonomic (e.g., relative motion) and comfort (e.g., usability quests). Nevertheless, a few studies are aimed at the assessment of physical interfaces. In recent years, physical interfaces have been assessed through kinematic and kinetic approaches, analyzing the interaction with the user. For instance, Pinto-Fernandez et al. [12] presented studies of ergonomic, interaction forces, and comfort found in the literature gathered 24 out of 187 articles addressing these topics. However, quantitative studies related to the assessment of physical interfaces have been overlooked. Hence, further analysis in this topic is required to ensure high-performance physical interfaces [12,22].

A few studies have been focused on quantitative indicators or experimental setups that assess physical interfaces, which are divided into two principal approaches: kinematic and kinetic analysis. These studies are resumed in Table 1.

On the one hand, Li et al. [16] presented the kinetic approach focused on the reaction forces between the physical interfaces and a passive exoskeleton. This study analyzed the kinetic differences, by allowing or restricting two degrees of freedom (DOF), as well as the internal forces and corresponding torques inside the physical interfaces. The variables are also measured indirectly along the sagittal plane, varying between 0.403 N and 20.678 N and 0.025 N.m and 1.949 N.m, respectively [16]. Another approach deployed by Leal-Junior et al. [26] embedded force sensors to identify the sinks of energy among the physical interfaces, in which the peak force varied between 5 N and 26 N. In this context, kinetic analysis quantifies the HRI on the physical interfaces; even so, a few studies have been deployed through more than one-dimensional (i.e., sagittal or transverse plane) approaches [24].

On the other hand, kinematic approaches are performed through reflective markers placed in the user and the device during a specific task (e.g., sit-to-stand or walking), acquiring the motion by an optoelectronic system [21,24,25]. These studies’ HRI indicators include multiple performance indicators such as interface displacements and adaptability to different height ranges. For instance, the ankle-foot orthosis presented by Langlois et al. [24] estimated the sagittal and frontal relative motion during walking of 16 mm and 11 mm, respectively. Regarding LLE, Akiyama et al. [25] presented a comparative motion analysis of sit-to-stand motions measuring a maximum physical interface’ displacement of 40 mm. Nevertheless, relative motion is only analyzed through displacement or rotation along the sagittal or transverse planes of the study, excluding the frontal plane and the analysis.

**Figure 1:**
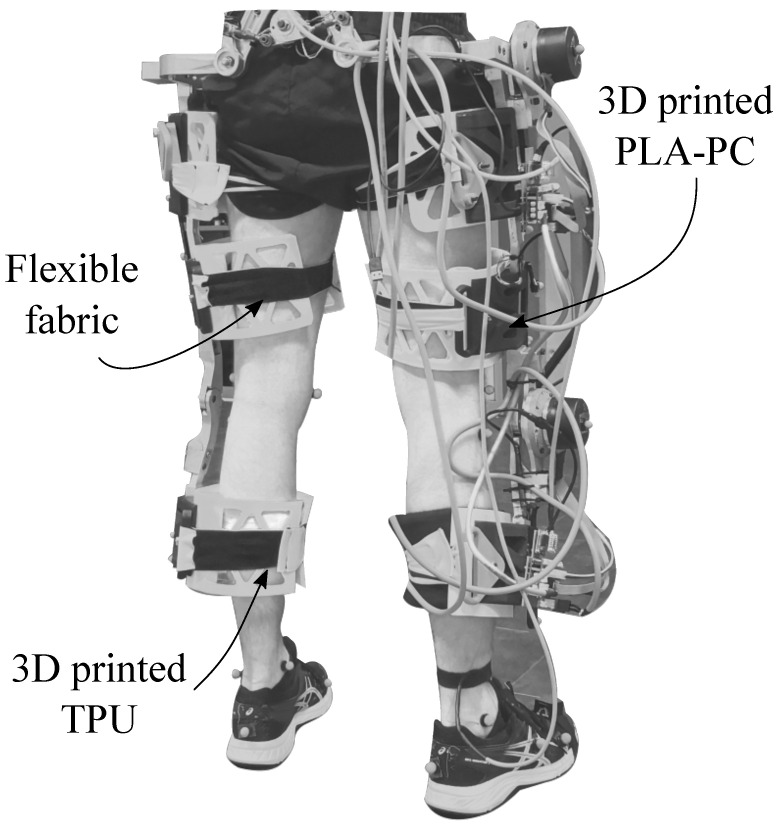
The AGoRA exoskeleton. Uni-lateral actuation on the right side.

Following the context, this work presents a novel three-dimensional relative motion analysis to fully assess the interaction between the user limb and the exoskeleton link. A preliminary study was carried out using the unilateral *AGoRA* (Adaptable Robotic Platform for Gait Rehabilitation) lower-limb exoskeleton (AGoRA-LLE), shown in Figure 1 [29]. The data analysis was divided into (1) descriptive statistics of the user’s interaction and (2) methodology consistency.

## 2. Materials and Methods

This section explains the proposed three-dimensional relative motion method, including the main principle and estimated interaction between the user’s limb and the exoskeleton. Likewise, this section also describes the experimental protocol used to implement and assess the interaction through the proposed method.

### 2.1. Three-Dimensional Relative Motion Method

**Figure 2:**
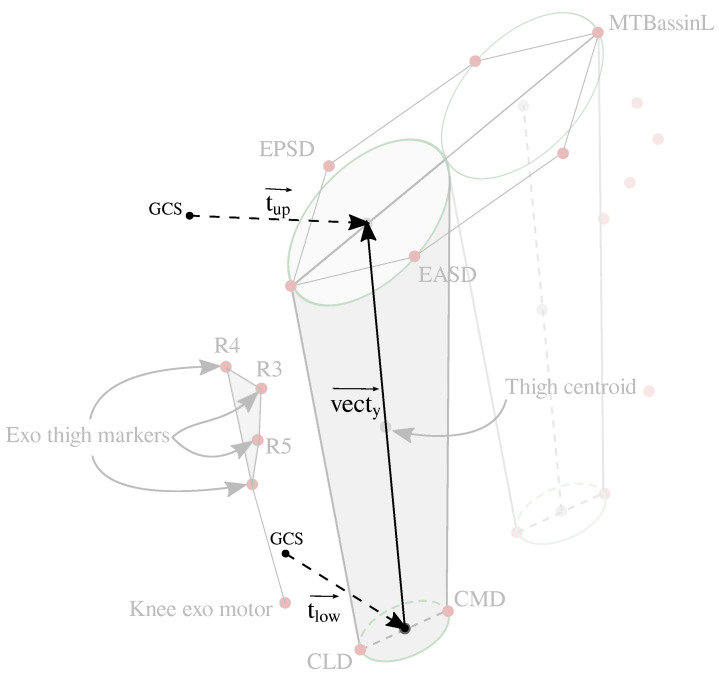
Scheme of thigh’s vectors. Reference vectors are used to establish the user’s local frame. The $\vec{t_{up}}$ vector is defined by the middle point between EPSD and EASD markers. Similarly, the $\vec{t_{low}}$ is created by the middle point between CLD and CMD markers. Finally, previous vectors are used to define the longitudinal vector of the thigh $\vec{vect_{y}}$.

A body’s motion can be described through a local frame that establishes its orientation compared to a global frame located at the origin [30]. In this lower-limb scenario, two bodies are defined through two local frames compared to the global frame. This analysis can be applied to any limb or joint. However, in this scenario, it is implemented in the assisted limb to comprehend the interaction between those bodies. The referenced points are defined through the markers set up and the global coordinate system (GCS), which are used to create multiple vectors (i.e., EPSD marker and the GCS to create the $\vec{OEPSD}$ vector). In this sense, the user’s references are established, as is shown in Figure 2.

The longitudinal vector of the thigh (i.e., $\vec{vect_{y}}$) is based on the $\vec{t_{up}}$ and $\vec{t_{low}}$ vectors as:

(1) $$\begin{array}{cc} \vec{t_{up}} & =\frac{1}{2}\cdot \left(\vec{OEPSD}+\vec{OEASD}\right) \end{array}$$

(2) $$\begin{array}{cc} \vec{t_{low}} & =\frac{1}{2}\cdot \left(\vec{OCMD}+\vec{OCLD}\right) \end{array}$$

(3) $$\begin{array}{cc} \vec{y_{th}} & =\vec{t_{up}}-\vec{t_{low}} \end{array}$$

Following, the medio-lateral vector is defined as the $\vec{t_{low}}$ and the external knee condyle as:

(4) $$\begin{array}{cc} \vec{z_{th}} & =\vec{OCLD}-\vec{t_{low}}, \end{array}$$

The anteroposterior vector ($\vec{x_{th}}$ is estimated as the cross product of $\vec{y_{th}}$ and $\vec{z_{th}}$, by definition pointing forward. Finally, the user’s thigh local frame is created by the unit vectors of $\vec{y_{th}}$, $\vec{z_{th}}$, and $\vec{x_{th}}$, which are used to define the user’s limb rotation matrix ($R_{th/O}$) compared to the global frame (*O*), representing the following unit vectors:

(5) $$\left[R_{th/O}\right]=\left[\begin{array}{c} \vec{X_{th}}\\ \vec{Y_{th}}\\ \vec{Z_{th}} \end{array}\right]=\left\{\begin{array}{cc} \vec{X_{th}} & =\frac{\vec{x_{th}}}{∥\vec{x_{th}}∥}\\ \vec{Y_{th}} & =\frac{\vec{y_{th}}}{∥\vec{y_{th}}∥}\\ \vec{Z_{th}} & =\frac{\vec{z_{th}}}{∥\vec{z_{th}}∥} \end{array}\right.$$

Similarly, the exoskeleton’s references also use three vectors which are defined by markers placed in the device’s structure and the physical interfaces. The longitudinal vector $\vec{y_{exo}}$ is defined as the unit vector between the two motor centers (i.e., right hip motor $exo_{HR}$, right knee motor $exo_{KR}$), pointing upward.

(6) $$\begin{array}{c} \vec{y_{exo}}=\vec{Oexo_{HR}}-\vec{Oexo_{KR}}, \end{array}$$

the medio-lateral vector $\vec{z_{exo}}$ is created through the cluster markers frame as a plane orthogonal to the vector, pointing laterally. This plane is defined by two vectors $\vec{v_{1}}$ and $\vec{v_{2}}$ as:

(7) $$\begin{array}{cc} \vec{v_{1}} & =\vec{OR_{3}}-\vec{OR_{4}} \end{array}$$

(8) $$\begin{array}{cc} \vec{v_{2}} & =\vec{OR_{3}}-\vec{OR_{5}} \end{array}$$

(9) $$\begin{array}{cc} \vec{z_{exo}} & =\vec{v_{1}}\wedge \vec{v_{2}} \end{array}$$

Then, the anterior-posterior $\vec{x_{exo}}$ is defined as the cross vector of $\vec{y_{exo}}$ and $\vec{z_{exo}}$, pointing upward.

(10) $$\begin{array}{cc} \vec{x_{exo}} & =\vec{y_{exo}}\wedge \vec{z_{exo}}, \end{array}$$

Finally, the exoskeleton local frame ($R_{exo/O}$) compared to the global frame (*O*) is defined through the previous vectors as:

(11) $$\begin{array}{cc} \left[R_{exo/O}\right] & =\left[\begin{array}{c} \vec{X_{exo}}\\ \vec{Y_{exo}}\\ \vec{Z_{exo}} \end{array}\right]=\left\{\begin{array}{cc} \vec{X_{exo}} & =\frac{\vec{x_{exo}}}{∥\vec{x_{exo}}∥}\\ \vec{Y_{exo}} & =\frac{\vec{y_{exo}}}{∥\vec{y_{exo}}∥}\\ \vec{Z_{exo}} & =\frac{\vec{z_{exo}}}{∥\vec{z_{exo}}∥} \end{array}\right. \end{array}$$

Using these two rotation matrices, (1) is defined as the orientation of the user’s thigh ($R_{th/O}$) placed in the knee joint, and (2) the exoskeleton’s thigh ($R_{exo/O}$) located in the knee hinge joint. These two matrices are compared to the global reference frame of the room. In the following, they are computed to explain the exoskeleton’s orientation compared to the user’s thigh ($R_{exo/th}$) as:

(12) $$\begin{array}{c} R_{exo/th}=R_{exo/O}R_{th/O}^{-1} \end{array}$$

**Figure 3:**
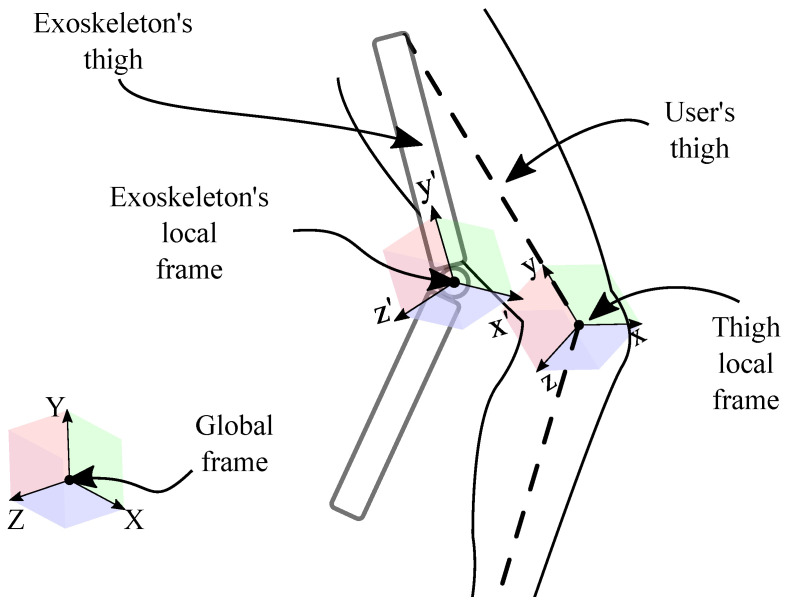
2D-projection of the descriptive scheme of rotation matrices.

The two local frames of the exoskeleton’s knee joint and user’s knee are summarized in Figure 3.

### 2.2. Experimental Protocol

#### 2.2.1. Subjects

All participants recruited for this study read and signed an experimental protocol and an informed consent in which they expressed their interest in participating voluntarily in this study. The experimental protocol, which included all the proposed procedures with the subjects, and the informed consent were approved by the local Ethics Committee of the Colombian School of Engineering. The volunteers were selected based on their health status and physical conditions, considering the inclusion and exclusion criteria, which are described below.

Males between 18 and 38 years old, with no history of neurological, neuromuscular, or physical disabilities that could affect their regular gait pattern, were included in the study. In addition, their height had to be within the range of 1.77 to 1.85 m, and their weight had to be less than 100 kg. Other inclusion criteria were some specific anthropometric measurements such as femur length between 42 and 48 cm, the distance between trochanters within the range of 32 to 37 cm, and tibial length between 28 and 31 cm. These limitations were established based on the functional range of the AGoRA-LLE, which can be adjusted.

On the contrary, volunteers were excluded from the study if they presented uncontrolled arterial hypertension, epilepsy, or if they were under the influence of alcohol, drugs, or any type of narcotic substance during the experimental procedure. Likewise, the volunteers were not considered in the study if they presented any cognitive impairment that prevented they from reading, understanding, or signing the informed consent form.

The study was conducted in a motion analysis room involving six healthy subjects considering the criteria described above. Their anthropometric characteristics are shown in Table 2.

#### 2.2.2. AGoRA Lower-Limb Exoskeleton

In this pilot study, the unilateral version of the AGoRA-LLE is used, whose overall weight is 12 Kg. For all volunteers, regardless of whether they are right- or left-handed, the exoskeleton is motorized only for the right limb, as shown in Figure 1. The actuation is performed in the sagittal plane for both hip and knee joints using an inertial measurement unit placed on the tip of the foot [31].

Additionally, the AGoRA-LLE has some mechanisms to ensure patient safety, such as: (1) a safety button that de-energizes the exoskeleton actuation system, (2) a mechanical limiting mechanism, and (3) an electronic limiting mechanism that limits the range of motion within normal gait parameters.

**Figure 4:**
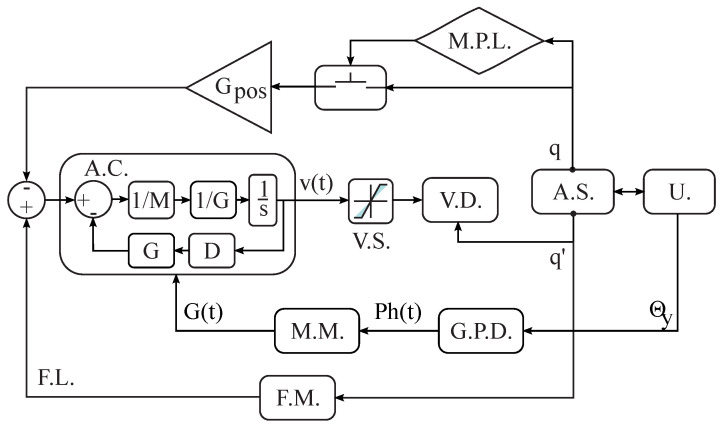
Controller scheme of the assistance mode. The admittance controller’ model is defined as a mass-damper system, including a module of position limitation, velocity saturation, and emergency button. These modules are aimed at the device’s security. (A.C.: Admittance Controller, F.L.: Feedforward Loop, F.M.: Friction Model, M.M.: Modulation Method, G.P.D.: Gait Phase Detection, U.: User, A.S.: Actuation System, V.D.: Velocity Driver, V.S.: Velocity Saturation, P.L.: Position Limitation, M.P.L.: Module of Position Limitation).

The thigh and shank variable links are adjusted for each subject according to the user’s anthropometric measurements, and the exoskeleton joints are manually aligning to the user’s hip and knee joints. The exoskeleton is initialized as assistance mode which is an admittance controller detailed in Figure 4 [32]. The gains (i.e., *G* and *D*) are adjusted for all the trials that allowed a resistive interaction to understand the response’s physical interfaces. This modality is configured for each subject and all its trials.

#### 2.2.3. Experimental Setup

**Figure 5:**
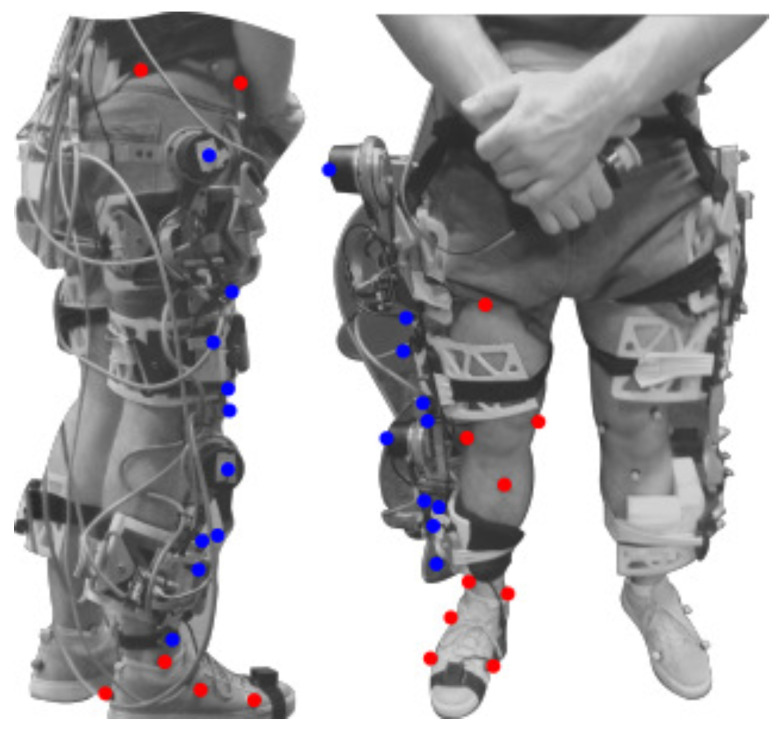
Markers setup used in the pilot study. Highlighted in red are the user’s markers and the exoskeleton’s markers in blue.

Kinematic data is acquired through 11 cameras (sampling frequency 100 Hz, accuracy 0.1 mm, Vicon Motion System, Oxford, UK) using 22 reflective markers (14 mm diameter) on the subject as described in Figure 5. The markers were distributed on the subject’s right leg: four markers at the physical interface of the lower thigh, one marker per motor joint (i.e., the hip and knee exoskeleton joint), and four markers at the physical interface of the shank. In this sense, the markers setup fulfilled the requirements to employ the 3D relative motion methodology as shown in Figure 5. Using this marker setup, the subjects performed ten trials of the 6-meter walking test. Moreover, it should be noted that both the markers and the exoskeleton were placed and instrumented only once; none of them were removed between sessions.

#### 2.2.4. Data Processing and Consistency Analysis

The methodology proposed for this preliminary study is divided and presented in two parts, first, (1) the methodology for analyzing user interaction and, second, (2) the methods for analyzing the consistency of the results obtained with the proposed novel three-dimensional methodology.

For the first analysis, once the labeling and gap-filling processing (i.e., the gap is understood as a zero value along a specific time span) is done in the NEXUS 2.9 (Vicon Motion Systems, UK) software, the .c3d files are exported to MATLAB (MathWorks, USA) programming environment. These files are read through the free Toolbox *Biomechanical ToolKit* (BTK) [33], which also allows the extraction of the different signals that describe the positions of the markers of interest.

Once all the information has been extracted, the signals are segmented into gait cycles based on the detection of the heel-strike event through the right heel’s reflective marker acceleration. To do this, one gait cycle is comprised of two consecutive heel-strike events performed by the same foot [34,35].

After the segmentation of cycles, the first and the last cycles were excluded due to signal instabilities caused by acceleration and deceleration in the gait. After ensuring stable gait cycles, they were normalized to facilitate the comparison between themselves. The previously proposed 3D relative motion analysis methodology is implemented by extracting the orientation signals. Each gait cycle corresponding to each orientation signal is analyzed throughout four gait phases, according to the percentage of the total gait cycle, e.g., flat foot at 10%, heel off at 50%, toe off at 73%, and heel strike at 100%.

The above process is performed for all *.c3d* files, segmenting and extracting all orientation signals (α, β and γ; or $Rot_{Z}$ relating the sagittal plane, $Rot_{X}$ relating the frontal plane, and $Rot_{Y}$ relating the transverse plane, respectively) through the rotation matrix $R_{exo/th}$.

Once these signals have been correctly segmented, it is necessary to analyze the differences between them for each subject. For this second analysis, also called the difference analysis, the mean, standard deviation, coefficient of variation, and cross-correlation values of the three orientation signals ($Rot_{X}$, $Rot_{Y}$, and $Rot_{Z}$) are calculated. The cross-correlation calculation allows measuring inter-subject variability; for the present case, it allows comparing each cycle of the orientation signal among themselves, establishing whether or not there is any similarity or homogeneity in the results. In most cases, the lack of homogeneity of the curves can be due to changes in the gait parameters or undesired movements of the mechanical structure throughout the tests.

**Figure 6:**
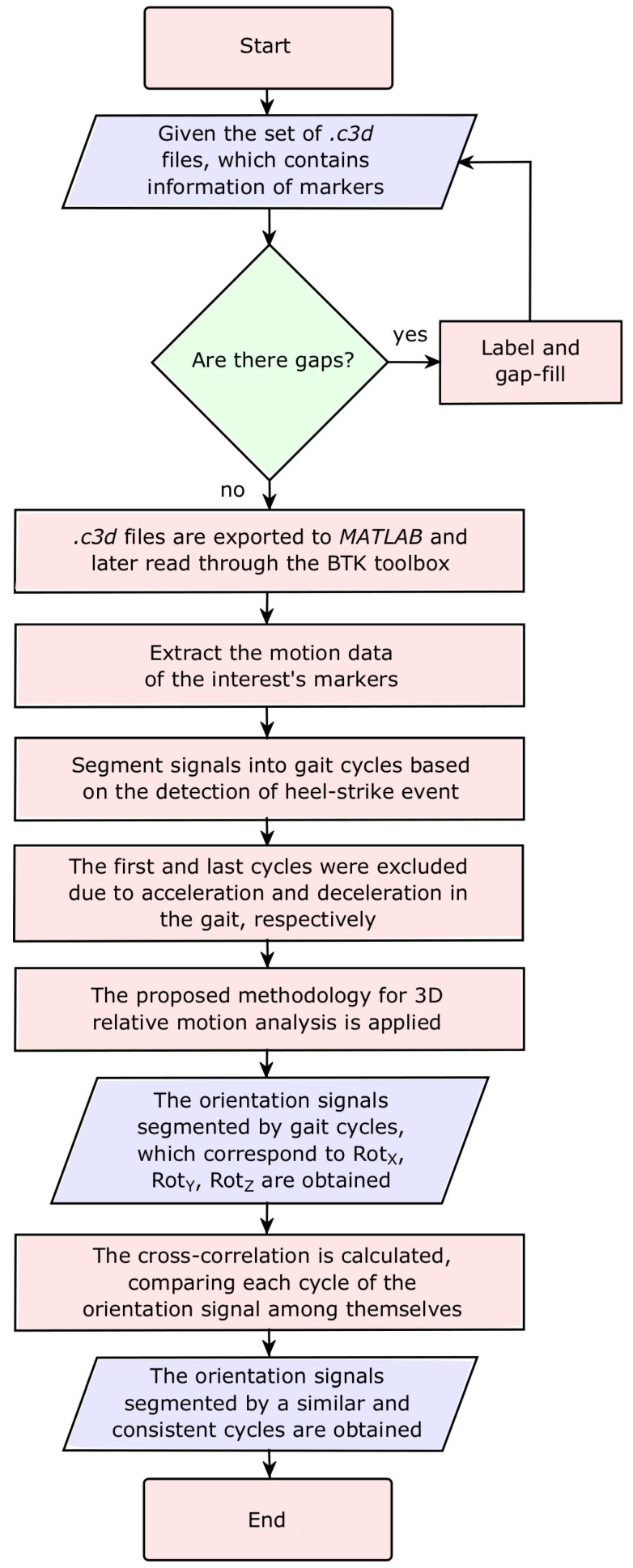
Flowchart of the data processing and implementation.

Figure 6 briefly describes the methodology implemented for data processing and consistency analysis.

Due to different factors, such as those mentioned in the previous paragraph, an initial misalignment of the frames could occur. Therefore, the offset had to be adjusted by subtracting the initial value of the misalignment, from which the different orientation changes were calculated.

## 3. Results

This study implemented the novel three-dimensional methodology, detailed in previous sections, which allows analyzing the user’s interaction through relative motion analysis. The results obtained in this preliminary study are divided and presented in two sections, first, (1) the results of user interaction, and second, (2) the results of the consistency of the methodology.

### 3.1. User’s Interaction

**Figure 7:**
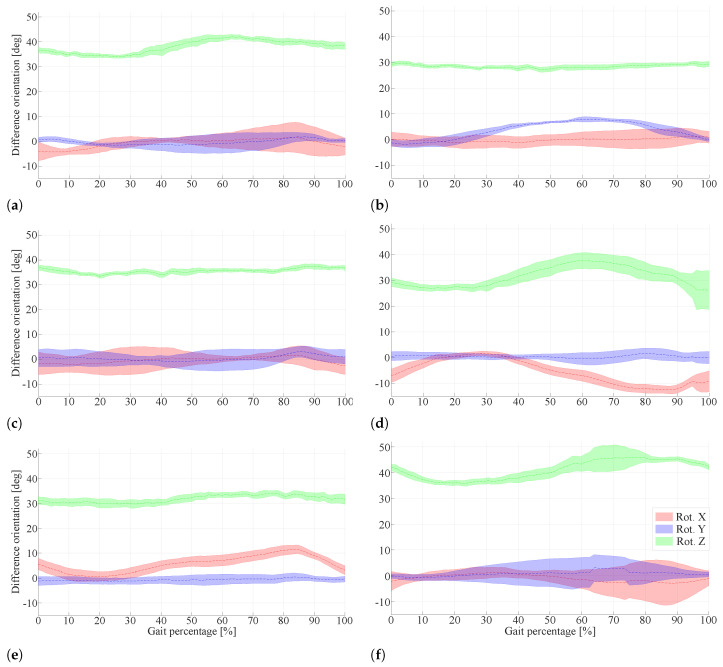
The difference of orientation’s outcomes per subject. (**a**) Subject 1, (**b**) Subject 2, (**c**) Subject 3, (**d**) Subject 4, (**e**) Subject 5, (**f**) Subject 6. The red curve refers to the $Rot_{X}$, the blue curve refers to the $Rot_{Y}$, and the green curve refers to the $Rot_{Z}$. In all cases, the dotted signal represents the average of the cycles for each orientation signal per subject; and the shaded curve represents the standard deviation of the cycles for each orientation signal per subject.

The results obtained related to the user interaction can be analyzed through the difference of orientations shown in Figure 7. These results are analyzed considering 21.3 ± 5.7 gait cycles per subject, for each orientation signal, $Rot_{Z}$, $Rot_{X}$, and $Rot_{Y}$.

As shown in Figure 7, the $Rot_{Z}$ presents a positive offset for all six subjects. Additionally, the difference of orientation corresponding to $Rot_{Z}$ varies within the range of 20${}^{\circ }$ to 45${}^{\circ }$. Particularly Subjects 2, 3, and 5 show a consistent behavior, in which there are no considerable increases throughout the entire gait cycle. However, Subjects 1, 4 and 6 present a different behavior, with an approximate increase of 10${}^{\circ }$ during the stance phase (i.e., between 0% and 73% of the total percentage of the gait cycle).

Something similar happened with the $Rot_{X}$. Although it presents negative or almost zero offsets, the difference of orientation corresponding to $Rot_{X}$ varies within the range of −15${}^{\circ }$ to 12${}^{\circ }$. For Subjects 1, 2, 3, and 6, $Rot_{X}$ presents a scatter behavior; but for Subjects 4 and 5, $Rot_{X}$ shows a sinusoidal shape and reduced scatter behavior compared to the other subjects.

Concerning the $Rot_{Y}$, it varies within −5${}^{\circ }$ to 12${}^{\circ }$ and presents an offset close to zero. In this case, the orientation difference is constant for five out of six subjects. For example, while Subjects 1, 3, and 6 show slight scatter behavior, the difference of orientation corresponding to Subject 2 varies in 10${}^{\circ }$ between 18% and 60% of the total percentage of the gait cycle.

Firstly, the overall mean is estimated in a total percentage of the gait cycle (i.e., percentage of the gait cycle from 0 to 100%), as shown in Table 3. This overall mean is calculated as the average orientation difference for all gait cycles per subject.

Considering the above, for all six subjects, the highest orientation difference is presented by $Rot_{Z}$ compared to the other rotations. The $Rot_{Z}$ range varies for a few subjects, representing a relative motion, although other subjects’ interaction showed a constant $Rot_{Z}$. The latter can be understood as an appropriate fixation to the user’s limb, even though the rotation difference will cause a loss of energy. Besides, the lowest orientation difference varies from subject to subject. For example, Subjects 1, 2, and 6 presented the most inferior orientation difference with $Rot_{X}$ close to zero. However, Subjects 3, 4 and 5 showed the lowest orientation difference with $Rot_{Y}$.

After these results, the analysis focuses on the range of rotation, calculating the mean, the standard deviation, and the coefficient of variation for each rotation and each subject, as shown in Table 3. Considering the range of rotation, the coefficient of variation was greater than 20% in all cases. Besides, five of the six subjects showed a coefficient of variation greater than 30% for each orientation, denoting a scatter behavior. The standard deviation remained below 4.5${}^{\circ }$ except for $Rot_{Z}$ for Subjects 4 and 6.

Another approach to understanding the interaction between the user and the exoskeleton is to consider the sub-phases throughout the gait cycle (i.e., heel strike at 0% or 100%, flat foot at 10%, heel off at 50%, and toe-off at 73%) [36]. Within these sub-phases, rotation ranges are also estimated, allowing analysis of the transitions in the same sub-phase. These results are shown in Table 4, wherein the heel strike, $Rot_{Y}$ showed a range of orientation closer to zero compared to $Rot_{X}$ and $Rot_{Z}$, showing an increase and decrease of 4.9${}^{\circ }$ and −5.0${}^{\circ }$, respectively.

The rotation ranges increase considerably in the flat foot compared to the first phase. Furthermore, 83.3% of the ranges showed a positive increase in physical interfaces during this same phase. This is the opposite of what happened in the third phase, where the range of rotation remained constant for Subjects 1, 2, and 3. Furthermore, for the other half of the subjects, it presented considerable changes in at least one of the rotations. Finally, the swing phase (i.e., from 73% to 100%) revealed the greatest decrease in the rotation range, and 66.6% were negative ranges.

### 3.2. Methodology’s Consistency

The cross-correlation is analyzed between the curves of the same rotation segmented by gait cycles per subject. The cross-correlation values can be between +1 and −1. A value of +1 means 100% correlation, while −1 means 100% correlation in phase opposition. A value of zero means that there is no correlation, and therefore the two signals are completely independent [37,38].

These results are presented in Table 5, where it can be seen that the $Rot_{Z}$ showed the most remarkable similarity (i.e., 0.97 to 0.99) for all the subjects. On the contrary, the $Rot_{Y}$ presented shallow cross-correlation values within a range of 0.03 to 0.12 except for Subject 4. Similarly, the $Rot_{X}$ also showed low cross-correlation values, within a range of 0.02 to 0.07 except for Subjects 4 and 5.

## 4. Discussion

The proposed methodology introduced new information regarding the HRI and the physical interfaces’ analysis. The relative motion is established through the proposed method which delved into the kinematic insight for this problem of exoskeletons. The methodology’s outcomes can be potentially used to improve the actuation performance, identify the transmission losses, and enhance the device’s design. On the one hand, the kinematic understanding can be used to adjust the device’s control strategies, knowing an estimation of the relative motion caused during the interaction. Besides, the relative motions also represent a loss of transmission, which can also be compensated within the control scheme. Hence, the HRI should improve according to these actuation adjustments. On the other hand, the interpretation of the results previously presented allows enhancing the device’s physical interfaces design. The undesired DOF are identified for the three anatomic human planes (i.e., sagittal, frontal, transverse), allowing one to understand the interaction and modify the physical interfaces to reduce or restrict these movements.

The results showed a higher variation of $Rot_{Z}$, which corresponds with the principal plane of the device’s actuation, causing an offset during the interaction. On the one hand, the $Rot_{Z}$ variation could be generated during the donning of the exoskeleton, establishing a physical offset of the placement between the user’s limb and the device’s link. On the other hand, the offset of $Rot_{Z}$ could be related to the compliance of the physical interfaces requiring lower compliance for adapting to the user’s body contours and adequately securing the device during its donning. Even though the physical interfaces’ compliance can be improved, they presented a constant response during the trials and the subjects, as shown in the consistency results.

Contrary to $Rot_{Z}$, the variations of $Rot_{X}$ and $Rot_{Y}$ showed inconsistent behavior between subjects. In addition to previously mentioned causes, these rotations are more susceptible to soft tissue artifacts due to the fixation layout of the exoskeleton (i.e., two braces in the thigh and one brace in the shank). As the fixation layout eases, the main motion along with the $Rot_{Z}$, at the same time, partially restricts the other rotations given that the soft tissue stretches and squeezes during the gait. These motions could be illustrated in the $Rot_{X}$ and $Rot_{Y}$ results. Nevertheless, the consistency of these rotations was low in most subjects, which could be explained by the variability between subjects.

Even though the exoskeleton is adjusted according to the anthropomorphic measurements of the user, the knee joint’s alignment varies between subjects. Assuming a proper donning of the device, the physical interfaces showed a rough interaction during the heel-off and the toe-off sub-phases for some subjects. This behavior happens for each rotation between the subjects. Even though these interactions are harmless for the subjects, they represent a transmission loss. In other cases, the physical interfaces allowed variations closer to 10${}^{\circ }$, which ease a comfortable interaction. Besides, it can be extended to displacement outcomes, instead of rotations and kinetic analysis. Following previous statements, the improvements of the physical interfaces are identified through the methodology.

The methodology can also be extended to displacement outcomes instead of rotations and kinetic analysis. The overall kinematic understanding of the body planes allows the estimation of interaction forces: moreover, undesirable forces are liable for energy losses. Besides, it can analyze the device’s donning through different scenarios and compare the knee’s misalignment.

As reported in the literature, these changes in the knee’s alignment affect the interaction, and they also involve other body planes [25]. This effect is shown in the user’s interaction results requiring more body planes than other studies. Current relative motion studies related to kinematic outcomes only analyzed translations along the sagittal plane [21,24,25]. Langlois et al. assessed a customized physical interface by estimating the distance between two markers in the sagittal and transverse planes along with the gait [24]. Even though the researchers examined the distance variation to the energy deployed by the exoskeleton, this analysis overlooked 33.3% of the interaction, which reduces the understanding of the energy losses in the task. Moreover, the physical interface’s response is only understood in two planes, minimizing the improvement for a future version of the physical interfaces.

Another relative motion study analyzed an LLE’s interaction during sit-to-stand along the sagittal plane. Akiyama et al. presented a deeper analysis of the thigh segment given two displacements and one rotation, as well as a physical interfaces’ slippage displacements [25]. In contrast to the proposed methodology, this allows us to fully understand the user’s interaction results, complementing the missed body planes, either frontal or transverse plane.

According to the above, previous studies showed a dimensional limitation to assess the relative motion during a specific task. Even though the methodology proposed to analyze three-dimensional relative motion, it also has a few limitations. This kinematic approach requires lateral markers to estimate the joints’ centers that define the local frame in the knee. These requirements might restrain the device’s implementation without any space between the limb and the structure. Nevertheless, multiple clusters could be solved in this issue, which provides other references to build these joint centers.

Another limitation of the proposed indicator, equally for other indicators found in the literature, is related to the mechanical response of the physical interfaces during the interaction. To ensure adequate interaction, the material’s analysis has to be included along the design process, taking into account the strain, adherence or stickiness, and deflection of the physical interfaces or defining proper compliance for these components according to the device’s targeted tasks. In this way, the physical interfaces maximize the transmission of energy to the user and reduce the slippage and undesired motions. However, currently, indicators do not assess or give an understanding of this phenomenon.

The pilot study also analyzes the kinematic compatibility involving different features such as adaptability, suitability, and comfortable [3]. Because of the HRI’s insight, these features are intended to be assessed among the studies of the AGoRA-LLE. The physical interfaces were evaluated through a usability test showing the main ergonomic issues to be improved [39]. Additionally, the three-dimensional analysis complemented the physical interfaces’ assessment. These outcomes quantified the HRI that provided an overall understanding of the physical interfaces’ response, allowing undesirable DOF along the user’s three principal planes. Therefore, this kinematic approach to quantify the HRI and evaluate the physical interfaces could enhance the device’s performance.

## 5. Conclusions and Future Work

This work presents a novel three-dimensional relative motion methodology to assess the AGoRA-LLE’s physical interfaces. An initial study was carried out by recruiting six volunteers and performed a 6-meter walking test. The AGoRA-LLE used an admittance controller for all the trials. The difference of orientation was calculated by the optoelectronic system using the reflective markers placed in the user and the exoskeleton. The proposed methodology provided a better understanding of the HRI and kinematic compatibility, analyzing the interaction in the three principal body planes.

The interaction estimated by the proposed methodology quantified a difference of orientation for $Rot_{Z}$ (i.e., along the sagittal plane) at a maximum value of 45 deg. It is also quantified a compensatory phenomenon regarding the difference of orientation echoed in the other rotations. In this sense, the proposed methodology fills kinematic gaps, shown in Table 1, regarding the variables and planes of study. Finally, the three-dimensional analysis explained physical interfaces’ response quantifying undesirable DOF that might represent losses of energy and discomfort.

This phenomenon can be seen through the coefficient of variation and the cross-correlation allowed for the identification of an accumulative effect in $Rot_{Z}$. These outcomes also extended the understanding of the relative motion analysis by 33.3 % and 66.6 % considering two more planes (i.e., $Rot_{X}$ and $Rot_{Y}$) than other works, as shown in Table 1. Besides, these planes showed considerable interaction with the overlooked planes. Moreover, the physical interfaces’ responses are also interpreted by the difference in the orientation of each rotation.

Further three-dimensional relative motion studies will be focused on the AGoRA-LLE physical interfaces enhanced version to reduce the difference of orientation. Further improvements will be related to the mechanical structure, aiming to increase the compliance of the device by adding soft components. In addition, the device’s controllers will also be compared to interpret the relative motion between them. Besides, the three-dimensional comparative motion analysis will be substantiated on a kinetic approach (i.e., torques, shear, and contact forces), extended to displacement outcomes, and linked to qualitative results, allowing for the definition of the quantitative range of comfort or discomfort. 

## Figures and Tables

**Table 1:** Quantitative studies of lower limb devices. The variables involved are one-dimensional displacement (D), one-dimensional rotation (R), force (F), torque (T), and power (P). The analysis is defined through gait (G), movement tasks (MT), and sit-to-stand (StS). The planes of study are divided into sagittal (S), frontal (F), and transverse (T). The *X* stands for including this variable or plane in the related study.

Author	Device	Task	Variable					Plane of Study		
			D	R	F	T	P	S	F	T
D’Elia et al. [21]	Pelvis orthosis	G	X	-	-	-	-	X	-	-
Langlois et al. [24]	Ankle-foot orthosis	G	X	-	-	-	-	X	-	X
Akiyama et al. [25]	Lower-limb exoskeleton	StS	X	X	-	-	-	X	-	-
Leal-Junior et al. [26]	Knee exoskeleton	MT	-	-	X	-	-	X	-	-
Rathore et al. [27]	REX	G	-	-	X	-	-	X	-	-
Li et al. [16]	Lower-limb exoskeleton	G	-	-	X	X	-	X	X	X
Yandell et al. [28]	Ankle-foot orthosis	G	-	-	-	-	X	X	-	-

**Table 2:** Table of characteristics of the subjects who participated in the study. (M.: Mean, S.D.: Standard Deviation).

Subject	Weight [kg]	Height [m]	Age [y.o.]
1	70	1.82	29
2	65	1.77	22
3	80	1.82	38
4	64	1.78	21
5	70	1.85	21
6	90	1.79	29
M. ± S.D.	73.17 ± 10.01	1.81 ± 0.03	26.67 ± 6.71

**Table 3:** Descriptive statistics of the difference of rotation. The overall mean presents the average value of each rotation for all the gait cycles. The rotation range is the difference between the maximum and minimum value for the each rotation’s mean curve. Units in degrees. (O.M.: Overall mean, M.: Mean, S.D.: Standard Deviation, C.V.: Coefficient of Variation).

Subj.	Rot.	Range of Rotation			
		O.M. [deg]	M. [deg]	S.D.	C.V. [%]
	*Z*	38.15	8.12	2.81	34.58
1	*X*	−0.72	5.34	3.26	61.04
	*Y*	1.11	3.55	1.76	49.60
	*Z*	28.45	2.57	1.57	61.08
2	*X*	−0.12	2.52	1.90	75.17
	*Y*	3.63	9.64	2.03	21.14
	*Z*	35.55	4.02	1.91	47.45
3	*X*	−0.32	4.26	2.87	67.47
	*Y*	0.22	5.96	2.10	35.29
	*Z*	31.52	11.50	8.79	76.07
4	*X*	−5.50	14.18	4.53	31.78
	*Y*	0.44	2.12	1.66	78.06
	*Z*	31.79	4.39	2.94	67.00
5	*X*	5.60	10.92	3.42	31.50
	*Y*	−0.67	1.73	1.90	110.12
	*Z*	41.10	10.17	5.83	57.34
6	X	−0.81	4.16	2.86	68.84
	*Y*	0.87	3.64	1.85	60.00

**Table 4:** Rotation range between gait sub-phases. The rotation difference is estimated according to the mean value at the corresponding gait percentage. Units in degrees. (M.: Mean).

Subject	Rotation	Difference of Rotation [deg]			
		Gait Percentage [%]			
		Flat Foot 0–10	Heel Off 10–50	Toe Off 50–73	Heel Strike 73–100
	*Z*	1.71	4.80	1.13	2.61
1	*X*	0.38	3.87	0.45	2.99
	*Y*	0.21	2.56	0.71	3.13
	*Z*	0.65	1.15	1.14	0.80
2	*X*	0.60	0.25	0.32	0.77
	*Y*	0.08	8.02	0.39	7.25
	*Z*	1.72	0.43	0.09	1.01
3	*X*	0.40	2.28	0.80	3.64
	*Y*	0.19	4.46	1.05	4.68
	*Z*	2.01	7.97	0.79	9.71
4	*X*	4.95	3.04	6.40	2.27
	*Y*	0.14	0.54	0.65	0.80
	*Z*	0.84	1.96	1.55	2.11
5	*X*	4.30	5.33	2.91	6.36
	*Y*	0.32	0.11	0.33	0.02
	*Z*	5.05	3.01	5.76	3.58
6	*X*	1.90	0.03	2.55	1.81
	*Y*	0.53	1.81	1.47	2.01
	*Z*	1.99	3.22	1.74	3.30
M	*X*	2.08	2.46	2.23	2.97
	*Y*	0.24	2.91	0.76	2.98

**Table 5:** Cross-correlation for the difference of rotations. Each group of curves is compared between them to analyze the similarity for each orientation.

Subject	Rotation		
	*Z*	*X*	*Y*
1	0.9969	0.0707	0.1108
2	0.9975	0.0172	0.8057
3	0.9977	0.0353	0.1251
4	0.9773	0.8482	0.0293
5	0.9946	0.8003	0.0521
6	0.9938	0.0305	0.0237

## Data Availability

Publicly available datasets were analyzed in this study, which is licensed under CC-BY 4.0. This data can be found here: https://doi.org/10.6084/m9.figshare.19372424 accessed on 20 February 2022.
